# A novel assay for measuring recombinant human lysophosphatidylcholine acyltransferase 3 activity

**DOI:** 10.1002/2211-5463.12712

**Published:** 2019-08-30

**Authors:** Xinming Du, Jiachun Hu, Qing Zhang, Qi Liu, Xinxin Xiang, Jibin Dong, Bin Lou, Shuhua He, Xiang Gu, Yu Cao, Yingxia Li, Tingbo Ding

**Affiliations:** ^1^ Department of Medicinal Chemistry School of Pharmacy Fudan University Shanghai China; ^2^ Department of Pharmacology and Biochemistry School of Pharmacy Fudan University Shanghai China; ^3^ Shanghai Science Research Center CAS Center for Excellence in Molecular Cell Science Shanghai Institute of Biochemistry and Cell Biology Chinese Academy of Sciences University of Chinese Academy of Sciences Shanghai China; ^4^ Institute of Precision Medicine The Ninth People's Hospital Shanghai Jiao Tong University School of Medicine China

**Keywords:** enzyme kinetics, inhibitor screening, LPCAT3, lysophosphatidylcholine acyltransferase 3, sequential mechanism

## Abstract

Lysophosphatidylcholine acyltransferase 3 (LPCAT3) is an important enzyme in phospholipid remodeling, a process that influences the biophysical properties of cell membranes and thus cell function. Multiple lines of evidence suggest that LPCAT3 is involved in several diseases, including atherosclerosis, non‐alcoholic steatohepatitis, and carcinoma. Thus, LPCAT3 may have potential as a therapeutic target for these diseases. In the present study, we devised an assay based on reversed‐phase HPLC to measure LPCAT3 activity, which may facilitate the identification of LPCAT3 inhibitors and activators. We found that optimal pH and temperature of recombinant human LPCAT3 are 6.0 and 30 °C, respectively. The enzyme *K*_m_ values for substrates NBD‐labelled lysophosphatidylcholine and arachidonoyl CoA were 266.84 ± 3.65 and 11.03 ± 0.51 μmol·L^−1^, respectively, and the *V*_max_ was 39.76 ± 1.86 pmol·min^−1^·U^−1^. Moreover, we used our new method to determine the IC_50_ of a known LPCAT inhibitor, TSI‐10. In conclusion, this novel assay can be used to measure the effects of compounds on LPCAT3 activity.

**Abbreviations**
Ara‐CoA: arachidonoyl coenzyme A
Bi–Bi: bisubstrate–biproduct; DDM; *N*‐dodecyl‐β‐d‐maltopyranoside
NBD‐lyso‐PC: NBD‐labelled lysophosphatidylcholine
NBD‐PC: NBD‐labelled phosphatidylcholine
rhLPCAT3: recombinant human lysophosphatidylcholine acyltransferase 3
SEC: size exclusion chromatography

Phospholipids are a major constituent in the cell membrane. The fatty acyl composition in phospholipids has an impact on the biophysical properties of the cell membrane and, consequently, the cell response to ambient stimuli. The majority of *de novo* synthesized phospholipids, which are composed of a saturated fatty acyl group in the *sn*‐2 position of the glycerol backbone, are matured by the Lands cycle, a remodeling pathway, in which the saturated fatty acyl group in the *sn*‐2 position is replaced by polyunsaturated acyl group upon the action of phospholipase A2 and lysophosphatidylcholine acyltransferase, respectively 1. Among the four isoforms identified so far, lysophosphatidylcholine acyltransferase 3 (LPCAT3) is of intensive interest because of its unique status in lipid metabolism. Expression of LPCAT3 is regulated by intracellular lipid homeostatic regulators such as PPARα, PPARδ and LXR 2, 3, 4, 5. LPCAT3 is the major isoform in liver, small intestine, adipose tissue and macrophages 6, 7, 8, 9. LPCAT3 activities determine the polyunsaturated fatty acyl composition in these tissues and cells. *Ex vivo* experiments have suggested that LPCAT3 activity is involved in small intestine tumorigenesis, macrophage polarization, hepatocyte apoptosis, adipocyte differentiation and adipogenesis 9, 10, 11, 12. *In vivo* experiments in mice indicate that LPCAT3 deficiency in small intestine has a dominant effect over its deficiency in the liver with respect to reducing plasma triglyceride and cholesterol levels because of the affected lipid absorption by small intestine 13. Mass spectrometry analysis in lesions and transplantation of LPCAT3 knockout hematopoietic cells into LDLR knockout mice suggest that LPCAT3 activity is associated with atherosclerosis 8, 14, 15. Followed by decreased LPCAT3, hepatocyte death mediated by the lipotoxicity effect of accumulated lyso‐PC may be the cause of non‐alcoholic steatohepatitis in mice fed a high‐fat diet with sucrose 11. Accumulating evidence suggests that pharmacological manipulation of LPCAT3 activity might provide novel therapeutic methods with respect to the pathological state of atherosclerosis, non‐alcoholic steatohepatitis, carcinoma, and immuno‐ and inflammatory disorders, etc. 16.

To screen compounds that regulate LPCAT3 activity, we purified a recombinant human LPCAT3 protein expressed in insect cells and then developed a reversed‐phase HPLC with a fluorescence detector method to quantify the catalytic product of this enzyme and determine its activity. By this method, we obtained the *K*_m_ of the substrate NBD‐labelled lysophosphatidylcholine (NBD‐lyso‐PC) and arachidonoyl coenzyme A (Ara‐CoA) under conditions of optimal pH and temperature. A sequential bisubstrate–biproduct (Bi–Bi) kinetic mechanism of this enzyme could also be proposed via Lineweaver‐Burk plots. Furthermore, we determined the IC_50_ of the LPCAT3 inhibitor in catalytic reactions with refined concentrations of enzyme and substrates, which could be applied to screen LPCAT3 regulators in the laboratory.

## Materials and methods

### Materials

NBD‐lyso‐PC/NBD‐labelled phosphatidylcholine (NBD‐PC) was purchased from Avanti Polar Lipids Inc. (Alabaster, AL, USA). Ara‐CoA was purchased from Sigma‐Aldrich (St Louis, MO, USA) NBD‐lyso‐PC/NBD‐PC/Ara‐CoA were dissolved in methanol and stored at −20 °C. BSA was obtained from Solarbio (Beijing, China). *N*‐dodecyl‐β‐d‐maltopyranoside (DDM) was purchased from Anatrace (Maumee, OH, USA). The bicinchoninic acid protein assay kit was purchased from Beijing ComWin Biotech Co. Ltd (Beijing, China). High‐performance Silica gel TLC plates (20 cm × 20 cm, 0.2‐mm gel thickness) were obtained from Yantai Jiangyou silicone development company (Shandong, China). TSI compounds were reported by Megumi Tarui as selective LPCAT2 inhibitors 17 and were synthesized by Xinming Du from the Department of Medicinal Chemistry of Fudan University (Shanghai, China). Chromatographic grade acetonitrile and methanol were purchased from Cinc High Purity Solvents Co. Ltd (Shanghai, China). Trifluoroacetic acid, chloroform and other regular reagents were purchased from Sinopharm Chemical Reagent Co. Ltd (Shanghai, China).

### Methods

#### Preparation and identification of the recombinant hLPCAT3

The coding cassette of human LPCAT3 gene (BC065194.1) was achieved by RT‐PCR with template RNA from cells of a human source before being cloned into pFAST‐Bac‐Rn plasmid with a Twin‐strep‐tag (WSHPQFEKGGGSGGGSGG‐SAWSHPQFEK) on the C terminus and a HRV 3C protease cleave site (LEVLFQ^↓^GP) between hLPCAT3 and the tag. The recombinant plasmid was named pFAST‐HC‐S‐hLPCAT3 and confirmed by sequencing. Bacmid and baculoviral of LPCAT3 were generated, and P3 viruses were used to infect Sf9 cells. Infected cells were harvested within 48 h and the membranes proteins were isolated: cells were suspended in 100 mL of low salt buffer (10 mm NaCl, 10 mm Hepes, pH 7.5, 1 mm phenylmethanesulfonyl fluoride) and then the pellet was collected after centrifugation at 45 000 ***g*** and 4 °C for 25 min. The pellet was resuspended in high salt buffer (1 m NaCl, 25 mm Hepes, pH 7.5, 1 mm phenylmethanesulfonyl fluoride, 5 mm MgCl_2_, 1 × Cocktail DNase) and homogenated until no visible particles could be seen. The mixture was centrifuged at 45 000 ***g*** and 4 °C for 25 min. Then, the pellet was further suspended in 50 mL lysis of buffer (150 mm NaCl, 20 mm Hepes, pH 7.5, 10% glycerol, 1 mm phenylmethanesulfonyl fluoride, 5 mm MgCl_2_, 1 × Cocktail DNase) and homogenated until no visible particles could be seen. After DDM was added to a final concentration of 1.5% w/v, the mixture was gently shaken for 2 h to help the membrane protein dissolve. The membrane protein was prepared in the supernatant after centrifugation at 45 000 ***g*** and 4 °C for 45 min. The recombinant hLPCAT3 protein was further purified from the membrane proteins using the Strep‐tag^®^ system (IBA GmbH, Göttingen, Germany): after the Strep‐Tactin^®^XT Superflow^®^ (IBA GmbH) resin was balanced successively with 10 mL of lysis buffer (150 mm NaCl, 20 mm Hepes, pH 7.5, 10% glycerol) and 10 mL of 2 mm DDM lysis buffer (150 mm NaCl, 20 mm Hepes, pH 7.5, 10% glycerol, 2 mm DDM), the supernatant containing membrane proteins was loaded onto the resin at a flow of 0.5–1 mL·min^−1^. The resin was washed twice with 10 mL of 2 mm DDM lysis buffer and then eluted using 3–4 mL of elution buffer (150 mm NaCl, 20 mm Hepes, pH 7.5, 10% glycerol, 2 mm DDM, 5 mm desthiobiotin). The eluate was further purified by size exclusion chromatography (SEC) (GE AKATA Pure^®^ system with a column of Superdex 200 Increase 10/300 GL; GE Healthcare Life Sciences, Chicdago, IL, USA). The mobile phase (150 mm NaCl, 20 mm Hepes, pH 7.5, 1 mm DDM) for SEC was set as a flow of 0.4 mL·min^−1^. Fractions of SEC that contain LPCAT activities were finally combined as the purified hLPCAT3 enzyme. The protein concentration was measured by the bicinchoninic acid method and the purity of this recombinant hLPCAT3 protein was identified by SDS/PAGE combined with Coomassie Brilliant Blue G250 staining. The activity of this purified recombinant hLPCAT3 was determined by the formation rate of the product NBD‐PC.

#### Isolation of liver microsomal proteins as the source of LPCAT3

Mice (10 weeks old) on a C57BL/6 background were purchased from Shanghai Model Organisms Center, Inc (Shanghai, China). Experimental mice were housed under a 12:12 h light/dark cycle in a temperature‐ and humidity‐controlled room. Mice were fed a chow diet. Experiments involving mice were conducted with the approval of Fudan University Institutional Animal Care and Use Committee. The procedures followed were in accordance with institutional guidelines.

Mice were sacrificed using cervical dislocation and liver tissue were dissected, weighed and then homogenized in 100 mm Tris‐Cl pH 7.4 containing protease inhibitor cocktail (Sigma‐Aldrich). After centrifugation for 10 min at 9000 ***g***, the supernatants were collected and centrifuged at 100 000 ***g*** for 1 h at 4 °C. The resultant pellets (microsomal proteins) were resuspended in 100 mm Tris‐HCl (pH 7.4) containing protease inhibitor cocktail (Sigma‐Aldrich) and stored at −80 °C. The concentration of the liver microsomal proteins was determined by the bicinchoninic acid method.

#### General enzymatic reaction of rhLPCAT3

A general enzymatic reaction of 100 μL was incubated at 30 °C for 10 min, which contained 1 mg·mL^−1^ BSA, 75 mm Tris‐Cl (pH 6.0), 1 mm DDM, 100 μmol·L^−1^ NBD‐lyso‐PC, 100 μmol·L^−1^ Ara‐CoA and one‐unit of purified recombinant human LPCAT3 (rhLPCAT3) enzyme. One‐unit of rhLPCAT3 activity was defined in this article as the amount of enzyme required to generate 25 pmol NBD‐PC·min^–1^ under the above conditions.

#### Analysis of NBD‐PC by TLC or reversed‐phase HPLC

For analysis of NBD‐PC by reversed‐phase HPLC, the reaction was terminated using 100 μL of acetonitrile. After vortexing for 15 s, the reaction mixture was centrifuged at 9000 ***g*** for 10 min and 20 μL of supernatant was used for the HPLC analysis. HPLC was performed with an HC‐C18 column (250 mm × 4.6 mm, 5 μm) (Agilent Technologies Inc., Santa Clara, CA, USA) and an isocratic elution with methanol/water/trifluoroacetic acid (94:6:0.04, v/v). The flow rate of the mobile phase was set as 1.0 mL·min^−1^. Detection was accomplished with a fluorescence spectrophotometer (excitation wavelength of 475 nm, emission wavelength of 525 nm). The photomultiplier tube gain was 12. All of the product NBD‐PC in the present study was measured by reversed‐phase HPLC.

For analysis of NBD‐PC by TLC, the reaction was terminated by adding 300 μL of chloroform/methanol (1:1, v/v) and vortexing. The lower organic phase was collected and dried under nitrogen gas after 15 s of vortexing and 10 min of centrifugation at 9000 ***g***. Residual lipids were redissolved in 40 μL of chloroform/methanol (2:1, v/v) and applied to a TLC plate, which was then developed using chloroform/methanol/H_2_O (65:25:4, v/v/v). The fluorescence signal was detected under UV.

#### Calibration curve of NBD‐PC

The calibration curve of the external standard method was used for the quantification of NBD‐PC. The calibration curve was constructed by plotting the peak area of product (NBD‐PC) (*y*) versus NBD‐PC concentration (*x*). The regression parameters of intercept, slope and correlation coefficient were calculated using prism, version 6.0 (GraphPad Software Inc, La Jolla, CA, USA). The concentration of NBD‐PC was calculated by a regression equation.

#### Time and enzyme concentration dependency of the recombinant hLPCAT3 catalyzed reaction

To investigate the enzyme concentration dependency, general reactions were carried out except for varied amount of purified recombinant hLPCAT3 from 0.03 to 2 μg·mL^−1^. Velocity of the reaction (pmol·min^−1^·U^−1^) was plotted against the concentration of the enzyme. To investigate time dependency, general reactions were carried out except for varied reaction time from 5 to 180 min. Stage of initial velocity was determined by plotting the velocity of the formation of NBD‐PC versus reaction time.

#### Effect of the pH and temperature on rhLPCAT3 enzyme activity

To investigate the effect of pH on the reaction, general reactions were carried out, except for a varied pH from 3.0 to 9.1. HCl or NaOH was used to adjust the pH of Tris‐Cl buffer. To investigate the effect of temperature on the reaction, general reactions were carried out, except for a varied temperature from 0 to −60 °C. Velocity of the reaction (pmol·min^−1^·U^−1^) was plotted against pH or temperature, respectively.

#### Characterization of the sequential kinetic Bi–Bi mechanism of the rhLPCAT3 reaction

To determine the *K*_m_ of NBD‐lyso‐PC and Ara‐CoA, general reactions were carried out except, for varied substrate concentrations. The kinetic mechanism and parameters of this Bi–Bi reaction were obtained by analyzing experimental Lineweaver‐Burk plots containing 16 velocity points. The substrate Ara‐CoA was held at several constant concentrations (0.75–9.00 μm) and the velocities were determined when the concentration of the substrate NBD‐lyso‐PC was varied from 20 to 100 μm.

#### Determination of the IC_50_ of the rhLPCAT3 inhibitor TSI‐10

Recombinant human LPCAT3 enzyme activity was measured to screen effective inhibitors. The inhibitory activities of different concentrations of the compounds were determined in a standard 100 μL reaction mixture containing 1 mg·mL^−1^ BSA, 75 mm Tris‐Cl (pH 6.0), 1 mm DDM, 11 μmol·L^−1^ NBD‐lyso‐PC, 11 μmol·L^−1^ Ara‐Coand purified rhLPCAT3 enzyme (one‐unit). The reaction was carried out at 30 °C for 10 min. Concentration of product NBD‐PC was analyzed by the HPLC and the IC50 of the inhibitors was calculated using prism, version 6.0.

### Statistical analysis

The analyzed data were expressed as mean ± SD. All statistical calculations were performed using Excel 2016 (Microsft Corp., Redmond, WA, USA) or prism, version 6.0.

## Results

### Recombinant hLPCAT3 preparation

**Figure 1:**
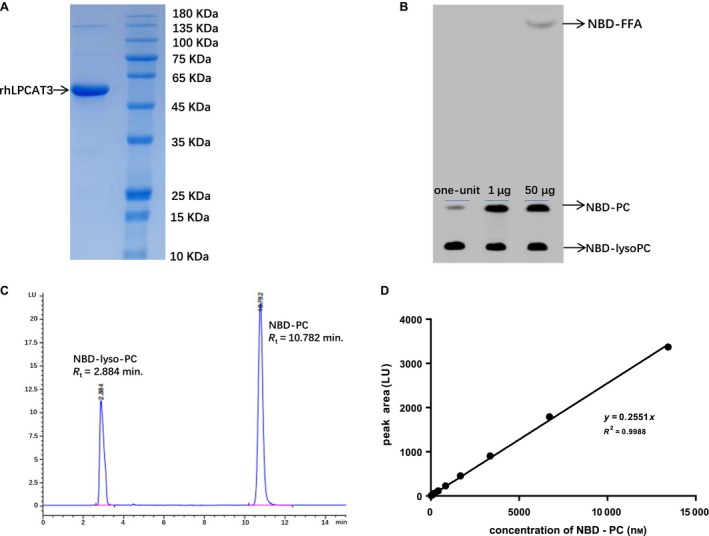
The purified recombinant hLPCAT3 showed properties enabling it to be an ideal tool for an activity assay by reversed‐phase HPLC. (A) rhLPCAT3 (60 kDa) identified by SDS/PAGE. (B) Activity comparison of rhLPCAT3 with liver microsomal proteins by TLC. From left to right: one‐unit of hLPCAT3 (0.0675 μg), 1 μg of rhLPCAT3 and 50 μg of liver microsomal protein in a 100 μL reaction system. (C) The retention time (*R*_t_) of NBD‐lyso‐PC (*R*_t _= 2.884 min) and NBD‐PC (*R*_t _= 10.782 min) in the HPLC analysis. (D) The calibration curve of the peak area (*y*) versus the concentration of NBD‐PC (*x*). The regression equation is *y* = 0.2551*x*,* r* ^2 ^= 0.9988.

To achieve a premium enzyme source, we expressed the hLPCAT3 protein in Sf9 insect cells and purified the protein by taking advantage of the fused twin‐strep tag in the C terminal of the rhLPCAT3. Analysis by SDS/PAGE revealed that a band of about 60 kDa, the calculated MW of rhLPCAT3, consisted of the major component of the purified protein, although a trace amount of contaminant protein remained after the purification process (Fig. 1A). The activity assay by TLC showed that the recombinant protein has a high specific activity of lysophosphatidylcholine acyltransferase because only 1 μg of the purified protein produced NBD‐PC identical to that obtained with 50 μg of microsomal proteins (Fig. 1B). Furthermore, the powerful phospholipase A1 activity that exists in microsomal proteins was removed by the purification process, as indicated by disappearance of the band of NBD‐labelled free fatty acid (Fig. 1B). Although we have a TLC method for LPCAT3 activity measurement, it is not sufficiently sensitive. We used the rhLPCAT3 to establish a new sensitive method for LPCAT3 activity analysis.

### The activity of rhLPCAT3 can be measured by reversed‐phase HPLC with fluorescence detector

**Figure 2:**
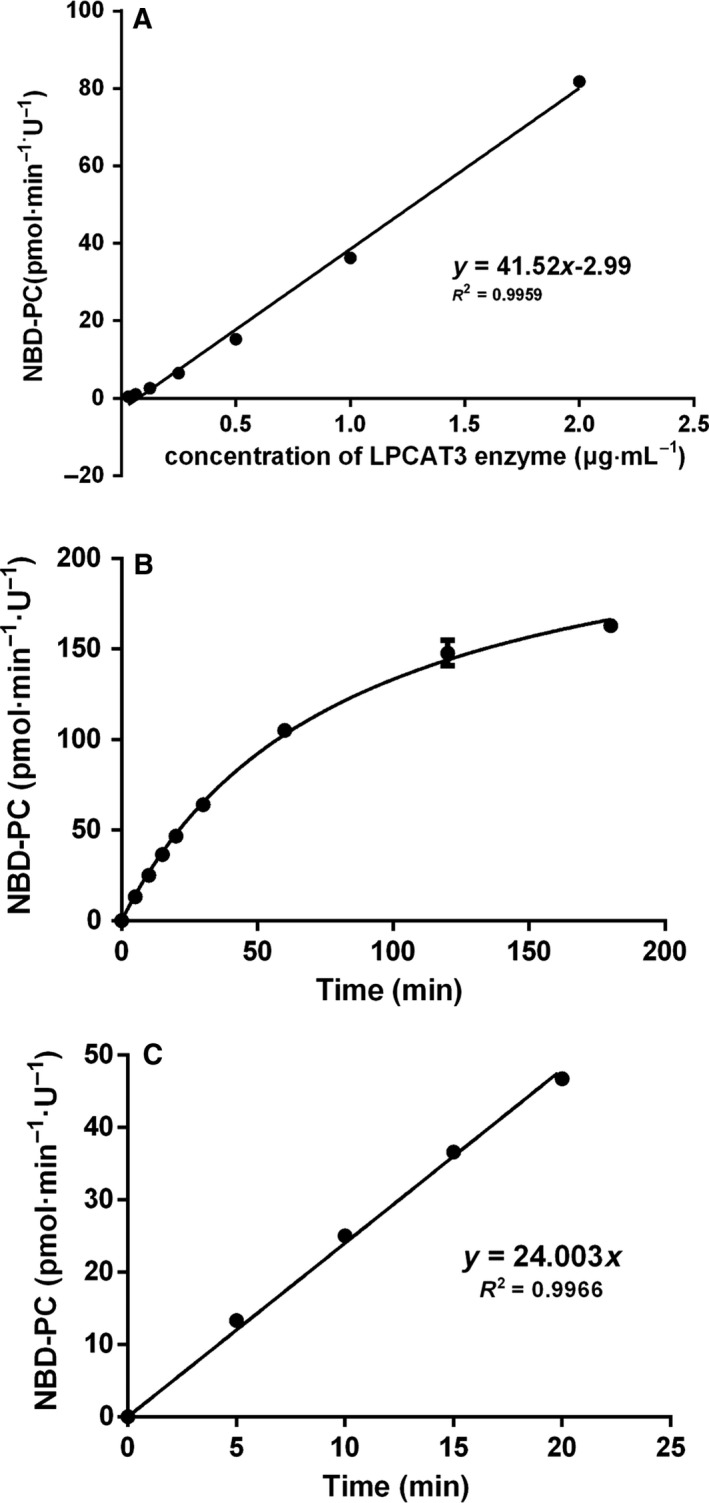
Enzyme concentration and reaction time dependent velocity analysis. (A) Velocity analysis under different amounts of rhLPCAT3 (from 0.03 to 2 μg·mL^−1^). The curve is linear and the correlation coefficient is *r* ^2 ^= 0.9959. (B) Velocity analysis of one‐unit of rhLPCAT3 incubated at 30 °C and pH 6.0 with different reaction times (from 5 to 180 min). (C) The stage of initial velocity is up to 20 min.

The enzymatic reaction product NBD‐PC could easily be detected by the fluorescence detector. We identified an optimal mobile phase with methanol/water/trifluoroacetic acid (94:6:0.04, v/v) for separating product NBD‐PC and substrate NBD‐lyso‐PC. The retention time is 10.782 min for the product NBD‐PC and 2.884 min for the substrate NBD‐lyso‐PC (Fig. 1C). The product signal could be measured within a linear range up to 13 433 nmol·L^−1^ NBD‐ PC (*r* ^2 ^= 0.9988) (Fig. 1D). To determine the activity of rhLPCAT3, we established a general catalytic reaction at pH 6.0, incubated at 30 °C for 10 min, using 100 μmol·L^−1^ NBD‐lyso‐PC and 100 μmol·L^−1^ arachidonoyl CoA in a 100 μL system. Different enzyme concentrations were used to investigate the enzyme concentration dependency on this condition. After mixing with equal volume of acetonitrile and centrifugation, the product NBD‐PC in 20 μL of supernatant could be analyzed by reversed‐phase HPLC. As shown in Fig. 2A, the formation of NBD‐PC catalyzed by rhLPCAT3 is linear (*r* ^2 ^= 0.9959) with the concentration of enzyme varying from 0.03 to 2 μg·mL^−1^, which means that the substrate concentration was sufficient to saturate the enzyme. To achieve uniform enzyme activity, we further defined one‐unit of rhLPCAT3 activity (0.675 μg·mL^−1^) as the amount of enzyme required to generate 25 pmol NBD‐PC·min^–1^ under general conditions.

To refine the catalytic reaction, the stage of initial velocity should be determined. Because one‐unit of the purified protein could produce sufficient NBD‐PC in the presence of 100 μmol·L^−1^ NBD‐lyso‐PC and 100 μmol·L^−1^ Ara‐CoA, we kept the reaction at 30 °C and pH 6.0 for varying times from 5 to 180 min (Fig. 2B). The results obtained showed that the amount of NBD‐PC increased at a fixed rate within 20 min, which indicated that all of the reactions in 20 min were in the stage of initial velocity (Fig. 2C). If only sufficient signal is produced for the HPLC measurement, a reaction time of 10 min was established for further experiments.

### Kinetic parameters of the rhLPCAT3

**Figure 3:**
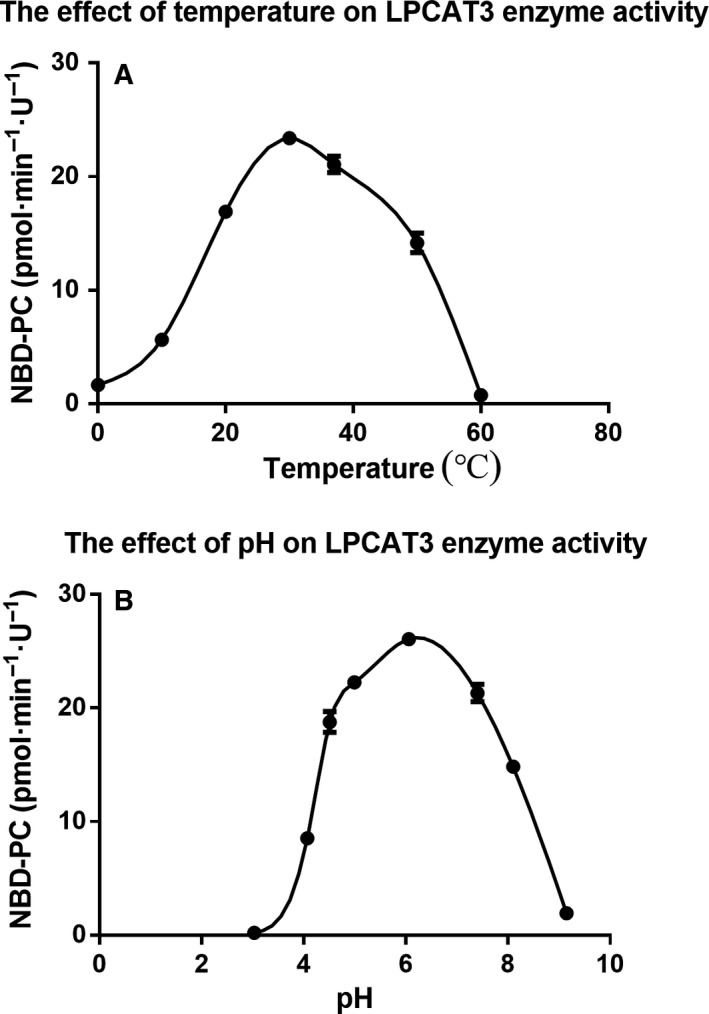
Optimal conditions for enzymatic reactions. (A) The effect of temperature on the activity of the rhLPCAT3, with an optimal temperature of 30 °C. (B) The effect of pH on the activity of rhLPCAT3, with an optimal pH of 6.0. Values are mean ± SD (*n* = 3).

**Figure 4:**
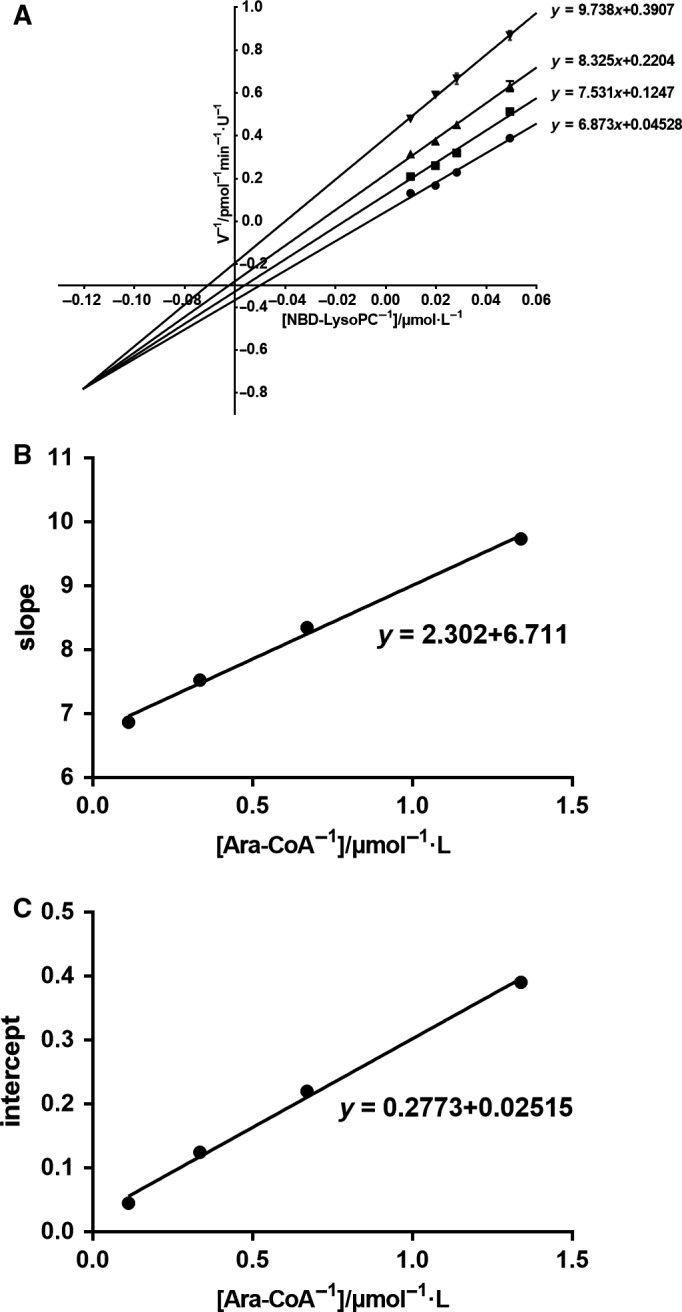
Determination of enzyme kinetic parameters and identification of the reaction mechanism. (A) The double reciprocal of initial velocity versus [NBD‐lyso‐PC] at several constant Ara‐CoA concentrations. (●) 9.00 μmol·L^−1^; (■) 3.00 μmol·L^−1^; (▲) 1.50 μmol·L^−1^; and (▼) 0.75 μmol·L^−1^. (B) Replots of slope as a function of [Ara‐CoA]^−1^. (C) Replots of intercept as a function of [Ara‐CoA]^−1^.

Provided that other factors were fixed, alteration of the rhLPCAT3 activity versus the pH was performed as shown in Fig. 3A, which revealed an optimal pH of 6.0. Similalrly, a change of rhLPCAT3 activity versus temperature was determined, as shown in Fig. 3B, which revealed an optimal temperature of 30 °C. Once the optimal pH and temperature were determined, we attempted to measure the _*K*m_ of NBD‐lyso‐PC and arachidonoyl CoA under this optimal condition. Because rhLPCAT3 used two molecules as its substrates, we calculated the *K*_m_ based on the Bi–Bi reaction mechanism. In the Lineweaver‐Burk plots where the rates observed with different fixed concentration of NBD‐lyso‐PC are plotted versus a series of constant concentration of Ara‐CoA, we found that the lines crossed at the same point on the left of 1/*v* axis, indicating a sequential mechanism rather than a ping‐pong mechanism in this Bi–Bi enzymatic reaction. We calculated the kinetic constants by the Dalziel formula, as widely used in bisubstrate reactions. We obtained a series of intercept and slope by double reciprocal plots of initial velocity versus [NBD‐lyso‐PC] at several fixed Ara‐CoA concentrations (Fig. 4A). Next, the Φ_NBD‐lyso‐PC_ and Φ_NBD‐lyso‐PC·Ara‐CoA_ were obtained by the plot of slope versus 1/[Ara‐CoA] (Fig. 4B), whereas Φ_Ara‐CoA_ and Φ_0_ were obtained by the plot of intercept versus 1/[Ara‐CoA] (Fig. 4C) (Φ_Ara‐CoA _= 0.2773, Φ_0 _= 0.02515, Φ_NBD‐lyso‐PC _= 6.711, Φ_NBD‐lyso‐PC·Ara‐CoA _= 2.302). The classical kinetic constants can be calculated by transforming the Dalziel equation to the Alberty equation: *V*_max_ = 1/Φ_0_ (39.76 pmol·min^−1^·U^−1^); *K*_m_ (Ara‐CoA) = Φ_Ara‐CoA_/Φ_0_ (11.03 μmol·L^−1^); *K*_m_ (NBD‐lyso‐PC) = Φ_NBD‐Lyso‐PC_/Φ_0_ (266.84 μmol·L^−1^) 18;

$$\text{Dalziel equation}:\frac{1}{V_{0}}=\Phi_{0}+\frac{\Phi_{B}}{\left[B_{0}\right]}+\frac{\Phi_{A}}{\left[A_{0}\right]}+\frac{\Phi_{\mathrm{AB}}}{\left[A_{0}]\cdot [B_{0}\right]}.$$

*V*_0_ is the initial velocity; [*B*_0_] is the initial concentration of Ara‐CoA; [*A*_0_] is the initial concentration of NBD‐lyso‐PC; Φ_0_, Φ_A_, Φ_B_ and Φ_AB_ are kinetic constants.

### Determination of the efficiency of certain rhLPCAT3 inhibitors

**Figure 5:**
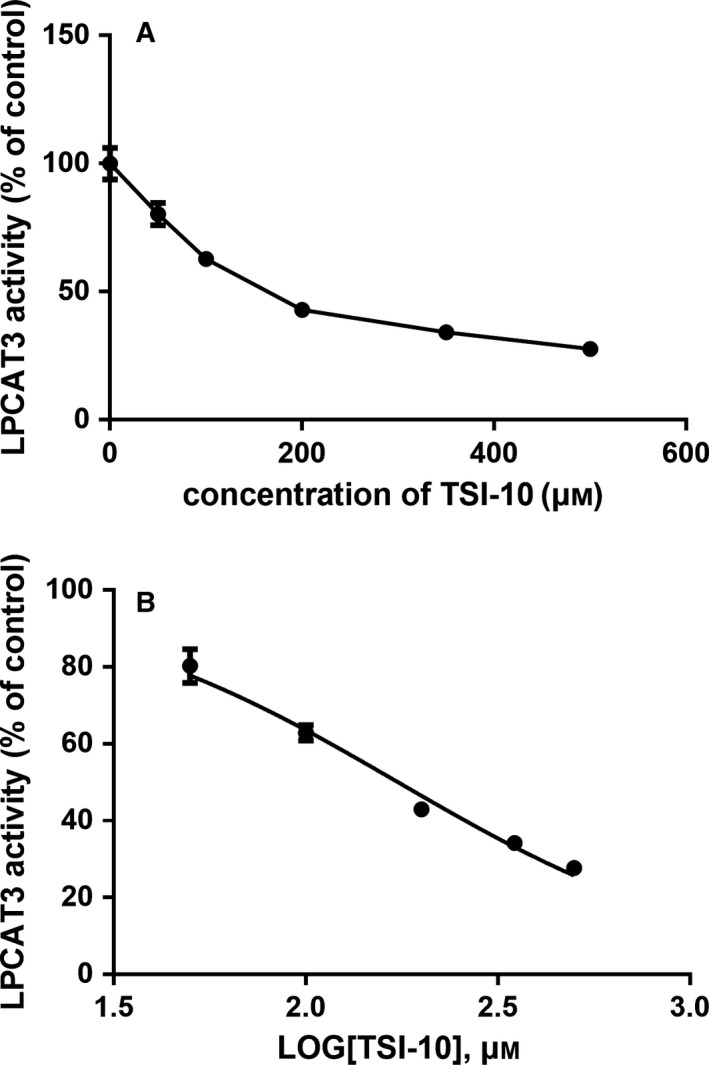
Determination of inhibitor IC_50_ by HPLC. (A) Representation of the inhibition studies with the reaction mixture containing one‐unit of rhLPCAT3, 11 μmol·L^−1^ NBD‐lyso‐PC/Ara‐CoA and a series of concentrations of TSI‐10. (B) The IC_50_ was calculated using prism, version 6.0. Values are the mean ± SD (*n* = 3). IC_50_ = 174.00 ± 1.04 μmol·L^−1^.

Because the HPLC method was established and the kinetic parameters were determined, we attempted to unify the catalytic reactions to measure and compare the power of rhLPCAT3 regulators. In reactions where factors such as activity of recombinant hLPCAT3 protein, substrate concentrations, optimal pH and temperature and reaction time were kept the same, the apparent rhLPCAT3 activities were measured versus a series of concentrations of candidate compound. Furthermore, the LPCAT2 inhibitor TSIs were screened to confirm the inhibitory activity on rhLPCAT3 by this method. However, only TSI‐10 showed inhibitory effects, with an example shown in Fig. 5A. The calculated IC_50_ value is shown in Fig. 5B.

## Discussion

Subsequent to LPCAT3 being identified and cloned about 11 years ago, multiple evidence has been obtained revealing that, besides lyso‐PC, this enzyme preferred polyunsaturated acyl CoA, especially arachidonoyl CoA, as its favorite substrate 2, 19, 20. Although radioactivity labeled substrates or the LC/MS/MS analysis method could be used to determine the activity of LPCAT3, we have attempted to develop an easily performed alternative method, using fluorescent substrate NBD‐lyso‐PC to establish the catalytic reaction, followed by measurement of the product NBD‐PC separated by TLC 6, 7, 9. Lipids were usually separated by TLC in a normal phase pattern and a large amount of chloroform would be used as a reagent of development solvent. Chloroform can be harmful for health if researchers are not properly protected. Furthermore, performing TLC is labor consuming and the intensity of the target band on the plate could be measured in a semiquantitative way. By contrast, with the aid of HPLC machine, samples were loaded automatically and analyzed quantitatively, which provided a more accurate, sensitive and labor saving method. In practice, HPLC is more widely accepted in the reversed‐phase pattern than in the normal phase pattern. Accordingly, when calculating the exact power of LPCAT3 regulators, we further developed a reversed‐phase HPLC with a fluorescence detector to measure the product NBD‐PC.

For screening compounds that regulate LPCAT3 activity, it is unsatisfactory to use tissue homogenate or microsome as the source of the enzyme because a large amount of substrate lyso‐PC would be consumed rapidly by a powerful phospholipase A1 activity that exists in the endoplasmic reticulum to produce free fatty acid, with NBD labeled in this case (Fig. 1B). Jain *et al*. 19 had attempted to express the human LPCAT3 in insect cells and harvest powerful activities from the cell homogenate 19. We also expressed the human LPCAT3 in this Sf9 insect cells, with a Twin‐Strep tag being fused with the C terminal of the target protein for further purification. Although a trace amount of contaminant proteins was not removed, the purified recombinant hLPCAT3 showed no phospholipase A1 activity, with a high specific activity to produce NBD‐PC. These properties suggest that the recombinant hLPCAT3 is an ideal tool for *in vitro* compound screening.

Because the combination of commercially available 12:0 NBD‐lyso‐PC with arachidonoyl CoA (when compared with palmitoyl CoA or oleoyl CoA) enables recombinant hLPCAT3 with the highest catalytic power (data not shown), we chose 12:0 NBD‐lyso‐PC and arachidonoyl CoA as the two substrates for a further rhLPCAT3 activity assay to produce a product of NBD‐PC with a NBD‐labeled dodecyl group (12:0) in the *sn*‐1 position and arachidonoyl group (20:4) in the *sn*‐2 position. The two substrates were dissolved in the methanol and could easily be distributed in the reaction buffer containing 1 mg·mL^−1^ BSA uniformly. This product NBD‐12:0 20:4 PC (retention time = 10.782 min) was easily separated from the substrate NBD‐lyso‐PC (retention time = 2.884 min) by reversed‐phase HPLC with a mobile phase of methanol/ddH_2_O/trifluoroacetyl acid (94:6:0.04, v/v). A higher methanol ratio in the mobile phase would cut down the retention time of NBD‐PC and save machine time for HPLC analysis, although at the cost of shortening the life span of the C18 column. The molar mass of NBD‐PC was determined by the fluorescence signal presenting a peak area within the linear range as high as 3300 LU (Fig. 1D).

When substrate and enzyme concentrations were fixed, the product NBD‐PC is linearly proportional to the reaction time up to 20 min (Fig. 2B,C). This suggested that all of the reactions within 20 min proceeded at the initial velocity. A 10 min reaction time was unified in further experiments. When substrates are fixed as 100 μmol·L^−1^ NBD‐lyso‐PC and 100 μmol·L^−1^ arachidonoyl CoA, respectively, the product NBD‐PC is linearly proportional to an enzyme concentration as high as 2 μg·mL^−1^ protein (Fig. 2A). This result suggested that recombinant hLPCAT3 enzymes up to 2 μg protein·mL^−1^ were saturated by the substrates at the indicated concentration. Based on these data, a primary protocol of the recombinant hLPCAT3 at as little as 0.657 μg·mL^−1^ protein (one‐unit), NBD‐lyso‐PC 100 μmol·L^−1^ and arachidonoyl CoA 100 μmol·L^−1^ was settled to establish the catalytic reaction because the fluorescence signal output was sufficiently sensitive for quantitative analysis. Under this condition, we identified an optimal pH of 6.0 and an optimal temperature of 30 °C. Jain *et al*. 19 had also reported a five‐fold higher activity at 28 °C than at 37 °C, although they performed the reaction at pH 7.4. We further determined the *K*_m_ of NBD‐lyso‐PC and arachidonoyl CoA by the model of Bi–Bi reaction under optimal conditions. Because the apparent *K*_m_ of one substrate is affected by the concentration of another in the Bi–Bi reaction, we obtained theoretical *K*_m_ values of 266.84 μmol·L^−1^ for NBD‐lyso‐PC and 11.03 μmol·L^−1^ for arachidonoyl CoA by the Dalziel equation. The *K*_m_ value of arachidonoyl CoA is comparable with that reported by Zhao *et al*. 2, 19, with an apparent *K*_m_ of about 72 μmol·L^−1^ at 37 °C and pH 7.5, and also that reported by Jain *et al*. 2, 19, with an apparent *K*_m_ of 55.8 μmol·L^−1^ at 28 °C and pH 7.4, and an apparent *K*_m_ of 245 μmol·L^−1^ at 37 °C and pH 7.4. Regarding the *K*_m_ of the NBD‐lyso‐PC that we determined, a comparable *K*_m_ for 1‐palmitoyl lyso‐PC of 72.19 μmol·L^−1^ was also reported by Zhao *et al*. 2 under a reaction condition of 37 °C and pH 7.5. However, the theoretical *K*_m_ values calculated by Bi–Bi kinetic equations were more accurate than that calculated by the Michaelis–Menten equation because the apparent *K*_m_ of one substrate calculated by the Michaelis–Menten equation may be influenced by the concentration of another, whereas Bi–Bi kinetic equations could overcome this disadvantage. Furthermore, from the Lineweaver‐Burk plots where the rates observed with different fixed concentrations of one substrate Ara‐CoA are plotted versus a series of concentrations of another substrate NBD‐lyso‐PC, a sequential kinetic mechanism could be proposed because plots of 1/*v* versus 1/[NBD‐lyso‐PC] at various constant values of [Ara‐CoA] (Fig. 4A) and 1/*v* versus 1/[Ara‐CoA] at various constant values of [NBD‐lyso‐PC] both converge to the left of the 1/*v* axis (data not shown), excluding the ping‐pong mechanism, which is characterized by parallels. The sequential kinetic mechanism suggests that all substrates must bind prior to the release of any products. The ping‐pong kinetic mechanism indicates that the product of the first reaction step is released from the enzyme before the other substrate binds. However, we can only infer a sequential Bi–Bi kinetic mechanism from Fig. 4A. The random Bi–Bi sequential mechanism and the ordered ternary complex sequential mechanism and the Theorell‐Chance‐type mechanism cannot be distinguished by the initial‐rate method individually. This limitation arises from the insensitivity of initial‐rate kinetics to the occurrence of internal isomerization reactions. Therefore, the distinct order of two substrates binds to the enzyme and product release remains to be explored.

In our general catalytic reactions, the concentration of both two substrates was settled at 100 μmol·L^−1^. As far as arachidonoyl CoA was concerned, this concentration was about 10‐fold of its *K*_m_, which was sufficiently high for kinetic analysis of the rhLPCAT3 enzyme. However, 100 μmol·L^−1^ of NBD‐lyso‐PC more or less reaches its *K*_m_, which imposes an inevitable fault on the kinetic analysis. However, more NBD‐lyso‐PC was difficult to add because of the poor solubility of this substrate.

Because any change of the factors would have an effect on the signal output, it is essential to refine factors such as reaction time, concentration of substrates, and enzyme, pH and temperature to meet the requirements of the rhLPCAT3 activity assay or *in vitro* compound screening. We considered one‐unit of rhLPCAT3 activity in our laboratories as that producing 25 pmol NBD‐PC·min^–1^ at 30 °C and pH 6.0 under a concentration of NBD‐lyso‐PC of 100 μmol·L^−1^ and arachidonoyl CoA of 100 μmol·L^−1^, respectively. In this standard, the specific activity of different batches of enzyme source should be determined before one‐unit of LPCAT3 activity was maintained for all of the experiments, which is essential for achieving comparable data.

Once enzyme activity was kept constant for all reactions, the concentration of the substrates became the prime factor that affected the values of IC_50_ as far as inhibitors were concerned. We modified the concentration of the substrates in our inhibitor screening experiments, keeping both NBD‐lyso‐PC and Ara‐CoA as 11 μmol·L^−1^, which is equal to the *K*_m_ of Ara‐CoA. This modification not only met the requirement for sensitive analysis, but also prevented unnecessary reagent consumption.

## Conflict of interest

The authors declare no conflict of interest.

## Author contributions

YL, YC and TD conceived the project. XD, JH, BL, JD contributed to the design and coordination of experiments. XD, JH, QZ, QL, XX, SH, XG were directly involved in experimental data acquisition. JH and TD write the manuscript. All authors reviewed the results and approved the final version of the manuscript submitted for publication.
